# Modulation of PTEN by hexarelin attenuates coronary artery ligation-induced heart failure in rats

**DOI:** 10.3906/sag-1812-49

**Published:** 2019-06-18

**Authors:** Elvis AGBO, Donghai LIU, Meixiu LI, Roland-Osei SAAHENE, Liqiang CHEN, Junpeng ZHAO, Yiquan WANG, Guozhong TIAN

**Affiliations:** 1 Department of Human Anatomy, Histology, and Embryology, College of Basic Medicine, Jiamusi University, Jiamusi P.R. China; 2 College of Basic Medicine, Jiamusi University, Jiamusi P.R. China; 3 Department of Immunology, College of Basic Medicine, Jiamusi University, Jiamusi P.R. China

**Keywords:** PTEN, hexarelin, hypertrophy, infarction, heart, failure

## Abstract

**Background/aim:**

Hexarelin is a synthetic growth hormone-releasing peptide that exerts cardioprotective effects. However, its cardioprotective effect against heart failure (HF) is yet to be explained. This study investigated the therapeutic role of hexarelin and the mechanisms underlying its cardioprotective effects against coronary artery ligation (CAL)-induced HF in rats.

**Materials and methods:**

Rats with four weeks of permanent CAL, induced myocardial infarction, and HF were randomly separated into four groups: the control group (Ctrl), sham group (Sham), hexarelin treatment group (HF + Hx), and heart failure group (HF). The rats were treated with subcutaneous injection of hexarelin (100 µg/kg) in the treatment group or saline in the other groups twice a day for 30 days. Left ventricular (LV) function, oxidative stress, apoptosis, molecular analyses, and cardiac structural and pathological changes in rats were assessed.

**Results:**

The treatment of HF rats with hexarelin signiﬁcantly induced the upregulation of phosphatase and tensin homologue (PTEN) expression and inhibited the phosphorylation of protein kinase B (Akt) and mammalian target of rapamycin (mTOR) to significantly improve LV function, ameliorate myocardial remodeling, and reduce oxidative stress.

**Conclusion:**

These findings indicate that hexarelin attenuates CAL-induced HF in rats by ameliorating myocardial remodeling, LV dysfunction, and oxidative stress via the upmodulation of PTEN signaling and downregulation of the Akt/mTOR signaling pathway.

## 1. Introduction

Globally, cardiovascular diseases have been identified as the leading cause of morbidity and mortality with heart failure (HF) being the main culprit over the past two decades (1,2). After myocardial infarction, mimicked in this study by the effect of coronary artery ligation (CAL), a wide range of intrinsic pathological processes occur in the myocardium causing myocardial remodeling and cardiac dysfunction, which ultimately leads to HF and death (3–7). However, in the present world, the treatment of myocardial infarction and HF with pharmaceutical agents has not been wholly successful and heart transplantation has not been a feasible option due to the scarcity of organ donors (8). This has therefore led to an increase in scientific research aimed at elucidating mechanisms that underline the development and regulation of cardiac remodeling and dysfunction since they will be of extreme value in the prevention of heart failure and other cardiovascular diseases.

Ghrelin, a known growth hormone secretagogue secreted in the stomach, is an endogenous ligand of GHSR1a with cardioprotective actions (9). Hexarelin is a synthetic analog of ghrelin with similar cardioprotective effects (10). Since it is much more chemically stable, it has proved to be a promising alternative to ghrelin (10). Numerous studies have reported on its beneficial actions associated with the cardiovascular system including the prevention of cardiomyocyte apoptosis (11), improved cardiac output (12–14), antiatherosclerotic effects (15), and cardiac fibrosis suppression (16). It binds to both the growth hormone secretagogue receptor GHSR1a and the cardiac non-GHSR receptor CD36 to exert its cardioprotective effects (17). 

In normal cellular functions, the PI3K/Akt/mTOR pathway plays vital roles such as proliferation, migration, adhesion, invasion, protein synthesis, energy metabolism, autophagy, and prosurvival roles (18–20), and it has also been described as a classical cardioprotective pathway (21). 

Phosphatase and tensin homologue (PTEN) is a lipid phosphatase responsible for the dephosphorylation of phosphatidylinositol 3,4,5-trisphosphate [PtdIns-(3,4,5)-P3] to produce PtdIns-(4,5)-P2, and therefore plays the role of a PI3K/Akt antagonist (22) by negatively regulating the PI3K/Akt/mTOR pathway.

The loss of PTEN in *Drosophila* has been demonstrated to result in increased growth, whereas the overexpression of PTEN has been shown to decrease cell size and cell number (23). Moreover, in mouse cardiac muscle cells, PTEN deletion has been demonstrated to result in myocardial hypertrophy and is closely associated with the upsurge in Akt activity and the size of individual myocytes (24). These results are all consistent with the hypothesis that the downregulation of PTEN results in increased Akt activity and could lead to myocardial remodeling, which, according to the literature, can ultimately lead to HF and death. PTEN can therefore be a potential target in the prevention of HF (25). However, whether the modulation of PTEN signaling is involved in hexarelin-induced cardioprotection is still unclear. Therefore, in the present study, we investigated the therapeutic role of hexarelin and the mechanisms underlying its cardioprotective effects against HF by testing the hypothesis that hexarelin can modulate PTEN to effectively attenuate CAL-induced HF in rats.

## 2. Materials and methods

### 2.1. Animals

This study was approved by the animal research ethics committee of Jiamusi University (No. 216-JMSU) and all research procedures were strictly performed in conformity with the guidance and suggestions for care and use of laboratory animals, published by the Ministry of Science and Technology of the People’s Republic of China. A total of 96 male SD rats aged 10–12 weeks weighing between 210 g and 230 g were obtained from the Experimental Animal Center of Harbin Medical University, Harbin, China. The animals were maintained in a 22 ± 2 °C temperature-controlled room under a 12-h light/dark cycle and had free access to food and water. 

### 2.2. Drugs

Hexarelin (C47H58N12O6) was purchased from ProSpec (East Brunswick, NJ, USA). In the experiments, hexarelin was dissolved in saline to the desired concentration before the administration of the drug.

### 2.3. Preparation of CAL-induced HF model and experimental protocols

First, 18 SD rats were randomly selected to form the control group (Ctrl; n = 18), and the rest took part in the CAL surgical procedure. The HF model was induced via the permanent ligation of the left anterior descending (LAD) coronary artery as previously demonstrated (26). Briefly, the SD rats were anesthetized by intraperitoneal (ip) injection with 10% chloral hydrate (0.3 mL/100 g, ip) and ventilated with a constant-volume rodent ventilator. After left thoracotomy and pericardiotomy were performed, the LAD coronary artery was permanently ligated using a 7-0 silk suture at approximately 3 mm from its origin. A confirmation of a successful CAL was done by the presence of ST-segment elevation on an electrocardiogram (ECG)and the presence of a color change at the ischemic region. The heart was then positioned well in the intrathoracic space, an evacuation of air was manually performed, and then the muscles and skin were closed. Randomly selected rats for the sham group (Sham; n = 18) underwent the same surgical procedure except for ligation. Four weeks after the surgery, the surviving ligated rats were divided into the hexarelin treatment group (HF + Hx; n = 20) and the heart failure group (HF; n = 20). All the rats in the four groups were then treated for 30 days. HF + Hx rats were treated with a subcutaneous injection of hexarelin (100 µg/kg, sc, twice a day) (1), while the remaining groups were treated with an equal volume of saline.

### 2.4. Echocardiography

Echocardiography was performed on all the rats after the completion of the hexarelin and saline treatment. The SD rats were first anesthetized by intraperitoneal injection (ip) with 10% chloral hydrate (0.3 mL/100 g, ip) and an experienced echocardiographer that had been blinded to the 4 different groups of rat assignments recorded two-dimensional M-mode stable images of the parasternal long- and short-axis views using a Sonos 7500 (Philips) echocardiographic system with the help of a 12-MHz transducer. The following parameters were measured: left ventricular end-diastolic diameter (LVEDD) and left ventricular end-systolic diameter (LVESD), with left ventricular ejection fraction (LVEF) being calculated in real time. All the measurements were averaged from 3 consecutive cardiac cycles.

### 2.5. Tissue preparation

After the echocardiographic measurements were performed, the hearts were rapidly harvested and tissues from the noninfarcted regions of the left ventricles were cut in cross-section at the papillary muscle level. Portions of the samples were directly fixed in 10% phosphate-buffered formalin, dehydrated, and embedded in parafﬁn. The other portions were frozen in liquid nitrogen, stored at –80 °C, and later used for RT-qPCR and western blotting.

### 2.6. Heart hematoxylin and eosin (H&E) staining

The paraffin wax-embedded tissue samples were cut into 4-µm slices. They were heated in a 60 °C incubator, dewaxed, and stained with H&E. A pathologist assessed the sample slices from each group in a blinded fashion and scored them for the following parameters: acute myocardial necrosis or cell death, neutrophil inﬁltration, degeneration of cardiac muscles, and hemorrhage.

### 2.7. Assessment of cardiac fibrosis with Masson’s trichrome staining

For the assessment of collagen fiber deposition as a marker of cardiac fibrosis, 4-µm slices of paraffin wax-embedded tissue samples that had been heated and dewaxed were stained using Masson’s trichrome dye. An Olympus DM4000 light microscope was used to capture the images of the heart sections. The evaluation of the left ventricle interstitial fibrosis area (LV interstitial fibrosis area = collagen area in LV / total LV area) was done using Image-Pro Plus 6.0 imaging software.

### 2.8. Assessment of cell apoptosis with Hoechst 33258 staining

Slices of paraffin wax-embedded tissue samples with the size of 4 µm that had been dewaxed were stained using Hoechst 33258 stain by strictly following the manufacturer’s instructions. The slides were observed using an Olympus fluorescence microscope and apoptosis was measured using Image-Pro Plus 6.0.

### 2.9. Immunohistochemistry

Cardiac tissues were processed for immunohistochemistry to analyze the expressions of PTEN, p-PTEN, Bax, Bcl-2, and caspase-3 proteins. Briefly, 4-µm sections of formalin-fixed, paraffin wax-embedded tissue samples were first deparaffinized, rehydrated, and rinsed three times in phosphate-buffered saline (PBS). Blocking of endogenous peroxidase activity was achieved by incubation with 3% hydrogen peroxide for 15 min at 37 °C and then washing three times in PBS. The sections were incubated for 30 min at 37 °C in goat serum to block nonspecific binding and then washed three times in PBS. Incubation was performed at 37 °C for 2 h with the following antibodies: anti-Bcl-2 (AF6139; 1:50) antibody purchased from Affinity Biosciences (Cambridge, UK), anti-PTEN (ab31392; 1:500) and anti-p-PTEN (ab131107; 1:50) antibodies purchased from Abcam (Cambridge, UK), and anti-Bax (50599-2-Ig; 1:20) and Caspase-3 (19677-1-AP; 1:50) antibodies purchased from Proteintech Group (Chicago, IL, USA). After washing with PBS, the incubation of the sections was done with biotinylated goat antirabbit secondary antibody for 20 min and also with streptavidin-horseradish peroxidase (HRP) conjugated working solution for 15 min at 37 °C before rinsing again with PBS. The reaction was developed by 5 min of incubation with 3,3’-diaminobenzene (DAB) and counterstained for 30 s with Mayer’s hematoxylin. After rinsing and dehydration, the slides were mounted under a light microscope. Image-Pro Plus 6.0 imaging software was used for the density analysis of the images.

### 2.10. Measurement of cardiac superoxide dismutase (SOD) and malondialdehyde (MDA) levels

Homogenates were prepared from the myocardial tissues of the rats and the measurement of the levels of SOD activity and MDA was performed using kits from Nanjing Jiancheng Bioengineering Institute following the manufacturer’s instructions.

### 2.11. Western blot analysis

Equal amounts of protein extracted from cardiac muscle tissues with the use of a buffer were separated on SDS-polyacrylamide gels and were electrophoretically transferred to polyvinylidene fluoride membranes. After blocking the membranes with 5% nonfat milk, they were incubated overnight at 4 °C with primary antibodies for PTEN (ab31392; 1:500) and p-PTEN (ab131107; 1:500) purchased from Abcam (Cambridge, UK), Akt (2920; 1:2000); p-Akt (4060S; 1:2000), mTOR (2983; 1:1000), and p-mTOR (2971S; 1:1000) purchased from Cell Signaling Technology (Danvers, MA, USA); GAPDH (1:1000) purchased from Beyotime (Jiangsu, China); Bcl-2 (AF6139; 1:1000) and BNP (DF6902; 1:500) purchased from Affinity Biosciences (Cambridge, UK); and Bax (50599-2-Ig; 1:1000) purchased from Proteintech Group (Chicago, IL, USA). They were then incubated with HRP-conjugated secondary antibody and the immunoreactive bands were subsequently detected with a chemiluminescence kit. 

### 2.12. Reverse-transcription quantitative real-time PCR

Total RNA was extracted from the myocardial tissues using TRIzol reagent according to the manufacturer’s instructions and was then reverse transcribed into cDNA using the Takara Prime-Script RT reagent kit (Takara, Dalian, China) according to the manufacturer’s protocol. The primers used were synthesized by Shanghai Sangon Biological Engineering Technology and Services Co. (Shanghai, China) and are as follows: BNP, forward: 5’-TGATTCTGCTCCTGCTTT-TC-3’, reverse: 5’-GTGGATTGTTCTGGAGACTG-3’; β-MHC, forward: 5’-ATG-CTGGCACCGTGGACT-3’, reverse: 5’-TTAGGAGCTTGAGGGAGGACTT-3’; TGFβ-1, forward: 5’-AAGAAGTCACCCGCGTGCTA-3’, reverse: 5’-TGTGT-GATGTCTTTGGTTTTGTCA-3’; procollagen-I: forward: 5’-TGCCGTGACCT-CAAGATGTG-3’, reverse: 5’-CACAAGCGTGCTGTAGGTGA-3’; and β-actin, forward: 5’-GAGGCTCTCTTCCAGCCTTC-3’, reverse: 5’- AGGGTGTAAAA-CGCAGCTCA-3’. Reverse-transcription quantitative real-time polymerase chain reaction (RT-qPCR) for the quantiﬁcation of the gene expressions was performed using Takara SYBR Premix Ex Taq (Takara, Dalian, China). The expression of β-actin served as a control for the normalization of the expression of genes of interest. The calculation of relative gene expression was done using the 2–ΔΔCt method.

### 2.13. Statistical analysis

SPSS 24.0 for Windows (IBM Corp., Armonk, NY, USA) was used for the statistical analysis. Experimental data were expressed as mean ± standard deviation (SD). One-way analysis of variance (ANOVA) or Welch’s method followed by the LSD post hoc test when appropriate was used to assess the statistical significance of the differences between the various groups. P < 0.05 was considered to be statistically signiﬁcant.

## 3. Results

### 3.1. Hexarelin improved LV function in CAL-induced HF rats

**Figure 1:**
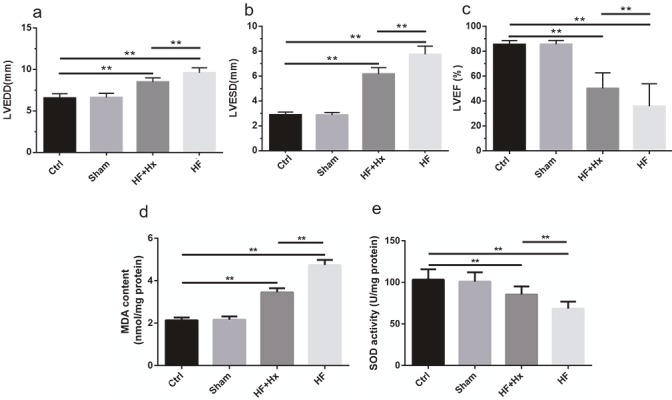
Echocardiographic results (a, b, c) showing the effects of hexarelin on CAL-induced HF rats with respect to: (a) LVEDD, (b) LVESD, (c) LVEF. Protective effects of hexarelin on CAL-induced HF rats against oxidative stress with respect to (d) malondialdehyde (MDA) and (e) superoxide dismutase (SOD). All data expressed as mean ± SD (n = 6). **P < 0.05.

As shown in Figures 1a and 1b, LVEDD and LVESD in the HF group significantly increased (P < 0.05) compared with the Ctrl group, indicating the enlargement of the heart and the impairment of diastolic and systolic functions. HF + Hx rats showed significantly decreased LVEDD and LVESD (P < 0.05) compared with the HF rats, suggesting that hexarelin ameliorates myocardial remodeling in CAL-induced HF rats. Figure 1c shows that there was a significant difference in LVEF between the HF group and Ctrl group (P < 0.05). The LVEF of the HF group was significantly lower than that of the Ctrl group. The LVEF of the HF + Hx group was significantly increased (P < 0.05) as compared to the HF group, which proves that hexarelin could improve the cardiac contractile function in CAL-induced HF rats.

### 3.2. The effects of hexarelin on the levels of SOD activity and MDA content

Compared to the Ctrl group, there was a significant increase (P < 0.05) in the MDA content (Figure 1d) accompanied by a significant drop (P < 0.05) in the level of SOD (Figure 1e) activity in the myocardium of HF group rats. The administration of hexarelin markedly reversed the decrease in the activity of SOD and the increase in MDA levels induced by CAL (P < 0.05). These results suggest that hexarelin exerts cardioprotective effects by playing an antioxidant role.

### 3.3. Hexarelin ameliorated cardiac structural and pathological changes in CAL-induced HF rats

**Figure 2:**
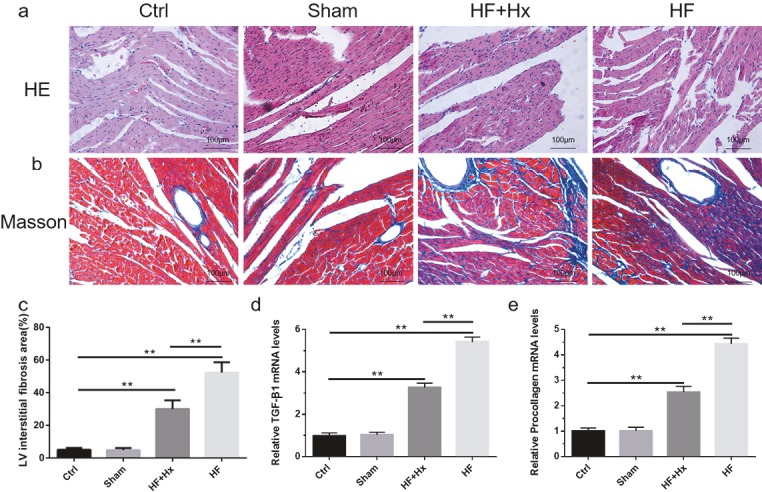
(a) Representative images of H&E staining of left ventricular sections (200×). (b) Representative images of Masson’s trichrome staining of left ventricular sections (200×). (c) Quantitation of left ventricular interstitial fibrosis area. (d, e) RT-qPCR analyses of TGF-β1 and procollagen-I. All data expressed as mean ± SD (n = 6). **P < 0.05.

Figure 2a shows the images of H&E-stained cardiac sections. Cardiac tissues in the HF group showed increased myocardial and morphological damages compared with rats in the Ctrl and Sham groups. These damages were characterized by myocardial cell loss and structural damage, loss of cross striations of myocardial fibers, myocardium fragmentation, rupturing, and inﬁltration of inﬂammatory cells (Figure 2a). The treatment of HF rats with hexarelin (HF + Hx group) conspicuously ameliorated the histopathological damages and pathophysiological changes in the myocardium of the HF + Hx rats.

### 3.4. Hexarelin attenuated collagen deposition and myocardial ﬁbrosis in CAL-induced HF rats

Figure 2b shows representative photomicrographs of Masson’s trichrome staining of noninfarcted left ventricular tissues. Red color indicates normal tissues while blue color indicates collagen fibers. The results of the present study show that there was a markedly significant (P < 0.05) increase in collagen deposition in the HF group compared with the Ctrl group. However, the treatment of HF rats with hexarelin (HF + Hx group) significantly attenuated (P < 0.05) the level of fibrosis or deposition of collagen although the difference between the HF + Hx group and the Ctrl group was still significant. We further calculated the LV interstitial fibrosis area in the cardiac tissues in order to accurately compare the differences in interstitial fibrosis level as well as cell survival among the groups. As shown in Figure 2c, the percentage of the LV interstitial fibrosis area was significantly elevated (P < 0.05) in the HF group compared to the Ctrl group. However, the HF + Hx group showed a significant decrease (P < 0.05) in the LV interstitial fibrosis area when compared to the HF group. These results clearly show that hexarelin attenuates collagen deposition and myocardial ﬁbrosis in CAL-induced HF rats.

### 3.5. The effects of hexarelin on fibrosis and cardiac hypertrophic remodeling-related biomarkers

**Figure 3:**
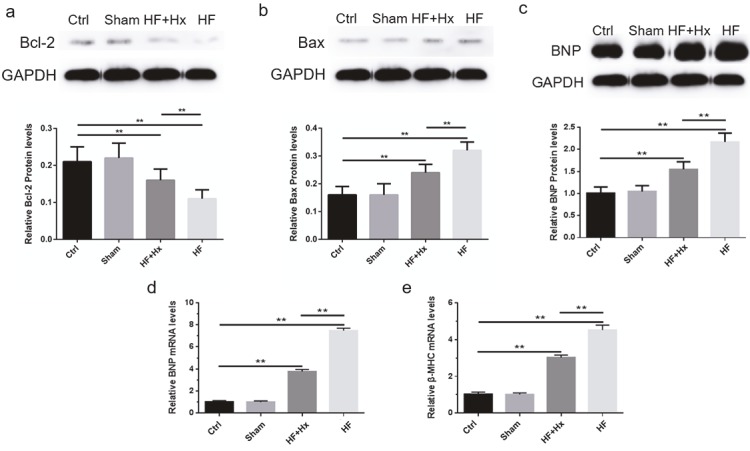
(a) Representative western blots and quantitation results of BNP. (b, c) RT-qPCR analyses of BNP and β-MHC. (d, e) Representative western blots and quantitation results of Bcl-2 and Bax. All data expressed as mean ± SD (n = 6). **P < 0.05.

Quantitative real-time PCR analysis revealed that the amounts of mRNA encoding transforming growth factor-β1 (TGF-β1) and procollagen-I were significantly increased (P < 0.05) in the HF group compared with the Ctrl group (Figures 2d and 2e). Upregulation in the expression of these genes was significantly inhibited (P < 0.05) in the HF + Hx group when compared to the HF group. Western blot analyses of the brain natriuretic peptide (BNP) (Figure 3a) as well as the mRNA expressions of BNP (Figure 3b) and β-myosin heavy chain (β-MHC) (Figure 3c) were all significantly elevated (P < 0.05) in the HF group as compared to the Ctrl group. Hexarelin therapy, as seen in the HF + Hx group, tended to significantly reduce (P < 0.05) all these vital cardiac hypertrophic remodeling parameters when compared to the HF group. These results in total suggest that hexarelin ameliorates myocardial remodeling-related cardiac hypertrophy and cardiac fibrosis in CAL-induced HF rats.

### 3.6. The effects of hexarelin on apoptosis

Our western blot results (Figure 3d) revealed that antiapoptotic protein Bcl-2 was significantly more highly expressed in the Ctrl group than in the HF group rats (P < 0.05) while the HF + Hx group showed a significantly increased expression of Bcl-2 (P < 0.05) when compared to the HF group.

The protein expression pattern of proapoptotic Bax was in sharp contrast with the Bcl-2 results. Bax (Figure 3e) was significantly highly expressed (P < 0.05) in the HF group rats as compared to the Ctrl group, indicating a severe increase in apoptosis during CAL-induced HF. However, the treatment of the HF rats with hexarelin significantly reduced (P < 0.05) the expression of Bax as seen when the HF + Hx group was compared to the HF group.

**Figure 4:**
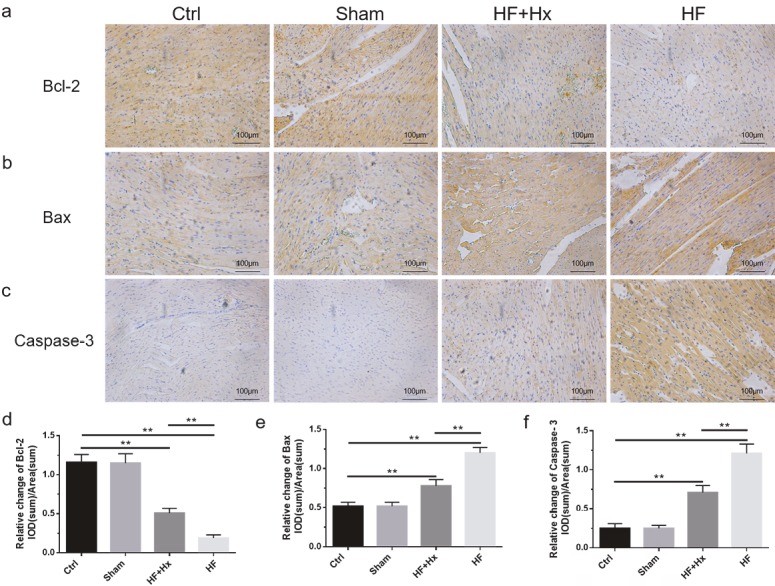
(a, b, c) Immunohistochemical expressions of Bcl-2, Bax, and caspase-3 in left ventricular sections (200×). (d, e, f) Quantitation of relative integral optical density (IOD) values of Bcl-2, Bax, and caspase-3. All data expressed as mean ± SD (n = 6). **P < 0.05.

**Figure 5:**
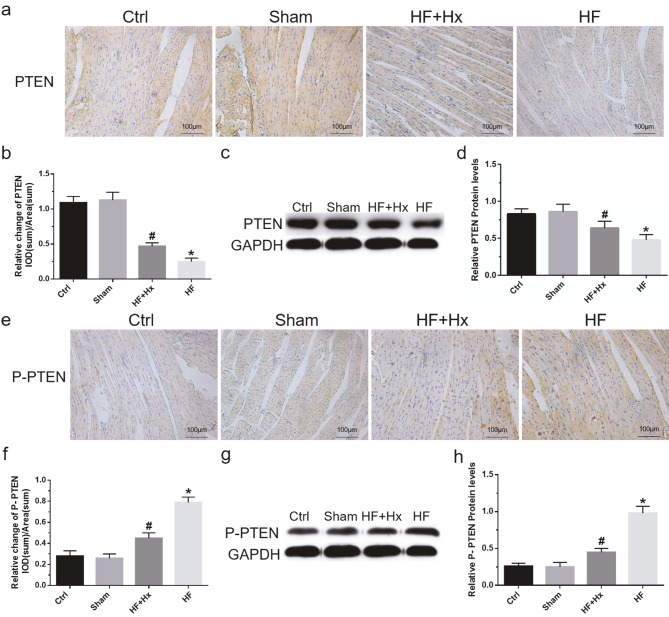
(a) Representative images of left ventricular sections showing the effect of hexarelin on apoptosis by Hoechst staining (200×). (b) Relative apoptotic index. All data expressed as mean ± SD (n = 6). **P < 0.05.

The immunohistochemical expression patterns of Bcl-2 and Bax (Figures 4a and 4b) were consistent with the western blot expression patterns of Bcl-2 and Bax proteins. Our immunohistochemistry results also showed that, compared to the Ctrl group, the HF group rats showed a significant increase (P < 0.05) in caspase-3 protein expression while the expression of caspase-3 (Figure 4c) was significantly decreased (P < 0.05) in the HF + Hx group compared to the HF group. Moreover, the quantitation of relative integral optical density values in our immunohistochemistry results (Figures 4d–4f) were all consistent with the immunohistochemical expression patterns of Bcl-2, Bax, and caspase-3 described above. In addition, Hoechst 33258 staining results (Figures 5a and 5b) revealed that the cardiomyocyte population showing apoptotic and condensed nuclei was significantly higher in the HF group compared to the Ctrl group, but was significantly reduced when the HF rats were treated with hexarelin (HF + Hx group). All these demonstrate that hexarelin attenuates apoptosis in CAL-induced HF rats.

### 3.7. The effects of hexarelin in the modulation of PTEN in CAL-induced HF rats

**Figure 6:**
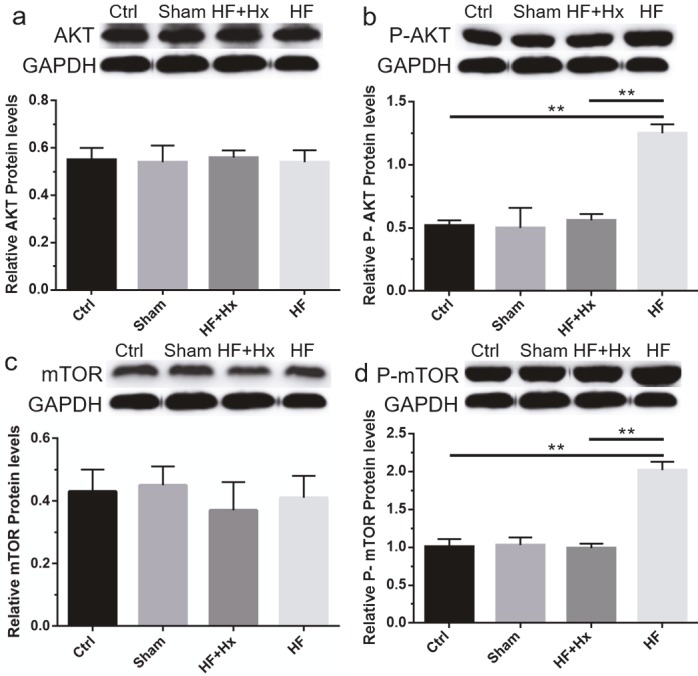
(a, b) Immunohistochemical expressions and quantitation of relative integral optical density (IOD) values of PTEN (200×). (c, d) Representative western blots and quantitation results of PTEN. (e, f) Immunohistochemical expressions and quantitation of relative IOD values of p-PTEN (200×). (g, h) Representative western blots and quantitation results of p-PTEN. All data expressed as mean ± SD (n = 6). *P < 0.05 with respect to HF versus Ctrl, #P < 0.05 with respect to HF + Hx versus HF.

Western blot and immunohistochemical analysis of PTEN were performed on cardiac tissue samples of rats in order to identify the mechanisms underlying the therapeutic role of hexarelin in attenuating CAL-induced myocardial remodeling and HF in rats. Active forms of PTEN are nonphosphorylated (PTEN) while inactive forms of PTEN are phosphorylated (p-PTEN). Compared to the Ctrl group, our immunohistochemistry and western blot results showed a significant decrease (P < 0.05) in the level of PTEN protein expression (Figures 6a–6d) with a corresponding significant increase (P < 0.05) in the p-PTEN protein expression in the HF group (Figures 6e–6h). Interestingly, the treatment of the HF rats with hexarelin (HF + Hx group) revealed a significant increase (P < 0.05) in PTEN with a corresponding significant decrease (P < 0.05) in the protein expression level of p-PTEN in the HF + Hx group when compared to the HF group. These results demonstrate that hexarelin upregulates PTEN activation by increasing the expression of PTEN with a corresponding decrease in the level of p-PTEN to attenuate CAL-induced myocardial remodeling and HF in rats.

### 3.8. The effects of hexarelin in the modulation of Akt/mTOR pathway-related proteins in CAL-induced HF rats

**Figure 7:**
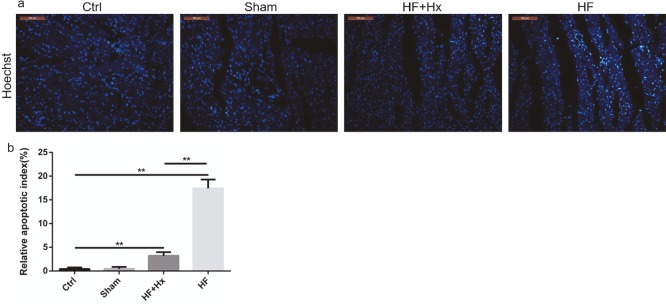
(a, b, c, d) Representative western blots and quantitation results of Akt, p-Akt, mTOR, and p-mTOR. All data expressed as mean ± SD (n = 6). **P < 0.05.

After the upregulation of the active form of PTEN by hexarelin, we further sought to investigate how hexarelin modulates the known downstream PI3K/PTEN-pathway components (Akt, p-Akt, mTOR, and p-mTOR) in CAL-induced HF rats. As demonstrated by western blotting (Figures 7a and 7b), the phosphorylation of Akt in the HF group was significantly enhanced (P < 0.05) as compared to the Ctrl group. However, compared to the HF group, there was a significantly marked decrease in Akt phosphorylation in the HF + Hx group due to the treatment of HF rats with hexarelin. We also examined the protein content of mTOR, a downstream target of Akt, by western blotting (Figures 7c and 7d). Expectedly, compared to the Ctrl group, our results showed a significant increase in the expression level of mTOR phosphorylation in the HF group. Interestingly, the increase in mTOR phosphorylation level in the HF group was reversed by the treatment of CAL-induced HF rats with hexarelin (HF + Hx group). These results show that hexarelin downregulates the phosphorylation of Akt and mTOR to attenuate CAL-induced myocardial remodeling and HF in rats.

## 4. Discussion

The main ﬁnding of this study is that the administration of hexarelin attenuates CAL-induced HF. Furthermore, we demonstrated for the first time that the mechanism of attenuation of HF by hexarelin is related to the upregulation of PTEN signaling and downregulation of the Akt/mTOR pathway. Our study also revealed that these beneﬁcial effects of hexarelin were due to its capacity to reduce oxidative stress as reﬂected by the increase in the activity of SOD and the decrease in MDA levels, the inhibition of apoptosis indicated by the decrease in the expression of proapoptotic protein (Bax) and apoptotic effector caspase-3 with a corresponding increase in antiapoptotic protein (Bcl-2) expression, and the attenuation of cardiac fibrosis and hypertrophic remodeling-related biomarkers in the myocardium of CAL-induced HF rats.

After myocardial infarction, a wide range of intrinsic pathological processes occur in the myocardium causing myocardial remodeling and cardiac dysfunction, which ultimately leads to HF and death (3–7). The present study used the CAL procedure, which is a classical operation method that is used to induce myocardial infarction in rats (27,28) and has been proven and accepted to be appropriate for the investigation of the pathophysiology of myocardial infarction and myocardial remodeling as well as HF in some rodents like mice and rats (26). 

Ventricular remodeling after a myocardial infarction is mainly characterized in the initial stages by cardiomyocyte death and an increase in inﬂammation in the periinfarcted and infarcted zones of heart. This is then subsequently followed by marked hypertrophy and fibrosis as well as the accumulation of prominent extracellular matrix (ECM) in the noninfarcted cardiac tissues in the later stages (29). Cardiac ﬁbrosis is a consequence of an imbalance between the processes in which ECM proteins are synthesized and degraded and is considered as a key process involved in the pathophysiology of heart failure (30). Collagen is a well-known fibrous protein and a major component of the ECM in heart. It is produced by fibroblasts. Moreover, its excessive deposition, which is known as pathological ﬁbrosis, is a major contributor to LV dysfunction and resulting HF (31–33). Our study demonstrated that hexarelin treatment markedly reduced the percentage of LV interstitial fibrosis area, indicating the antifibrotic ability of hexarelin, which in effect resulted in the improvement of cardiac contractile function showed by echocardiographic results. In addition, the mRNA expression level of procollagen-I, a biomarker of fibrosis that reflects the intensity of myocardial collagen synthesis (34–37), was significantly reduced by hexarelin treatment. TGF-β1, which belongs to the TGF-β family of proteins, is involved in the promotion of fibrosis in cardiovascular and renal tissues by increasing the production of ECM (38); hence, it is regarded as one of the molecular indices that regulate myocardial ﬁbrosis. As speculated, TGF-β1 mRNA expression level was also inhibited by the hexarelin treatment of HF rats. Cardiac hypertrophy has over the years been a potential target of anticardiac remodeling therapy and several pieces of evidence in humans are consistent with therapeutic targeting of the myocardial hypertrophic process (39). Although short-term cardiac hypertrophic remodeling is beneficial, its long-term activation is detrimental and pathological, leading to a progression towards HF. Therefore, these strongly suggest that the suppression of pathological cardiac hypertrophic remodeling may be vital in impeding its progression to HF (40). The characteristic pattern of protein and mRNA expressions classically related to pathological LV hypertrophy or remodeling involves increased BNP and β-MHC levels. Consistent with our results in this study, hexarelin markedly counteracted this characteristic pattern of pathological LV remodeling. All these results in totality strongly suggest that hexarelin ameliorates myocardial remodeling-related cardiac hypertrophy and cardiac fibrosis to improve cardiac contractile function in CAL-induced HF rats and in effect attenuates HF.

Oxidative stress is one of the mechanisms underlying myocardial damage (41). An increase in free oxygen radicals produced in the ventricles after MI results in lipid peroxidation that can indirectly be reflected through ventricular MDA level. In our study, we observed that the administration of hexarelin reversed the adverse increase in MDA levels and also raised the level of endogenous antioxidant SOD activity, which indicates strongly that the antiperoxidative capacity of hexarelin partakes in the protection of the heart to attenuate CAL-induced HF in rats.

Some previous studies reported that hexarelin could improve cardiac function (42) but the majority of them failed to elucidate the mechanism by which hexarelin exerts these cardioprotective functions. Therefore, our study sought to bridge this mechanism research gap. There has been accumulating evidence in several recent studies suggesting a cardioprotective role involving the PTEN gene (23,24,43–45). For example, an investigation on the cardioprotective effects of simvastatin on the expression of PTEN and the Akt signaling pathway revealed that simvastatin reverses cardiomyocyte hypertrophy, which is a characteristic feature of myocardial remodeling both in vitro and in vivo via the upregulation of PTEN expression and the downregulation of Akt (43). These results therefore improve the prognosis of HF. Roy et al. used a murine myocardial infarction model to demonstrate that miR-21 modulates ﬁbroblast metalloprotease-2 through PTEN (45). In their experiment, they showed that there was a significant decrease in PTEN expressions in the myocardial infarct zone of the heart as compared to that of the control. This suggested that an upregulation of PTEN could be beneficial and cardioprotective. Fu et al. also showed that attenuation of microRNA-495 activates PTEN to effectively protect cardiomyocytes of rats from pathological cardiac hypertrophy or remodeling (44), which will ultimately lead to an approach in the attenuation of HF occurrence. Interestingly, our western blot and immunohistochemical analyses in the present study demonstrated that hexarelin upregulates PTEN activation by increasing the expression of PTEN with a corresponding decrease in the level of PTEN phosphorylation to attenuate CAL-induced myocardial remodeling and heart failure in rats, which strongly indicates that PTEN is associated with the cardioprotective effect of hexarelin. 

Although our results were consistent with the vast majority of evidence supporting the protective role of PTEN in heart failure, a study conducted by Oudit et al. in 2008 showed that the loss or inhibition of PTEN may help to prevent ventricular hypertrophy (46). This discrepancy can be explained by the existence of different experimental models or protocols. In other words, PTEN might be modulated differently depending on the cause of the resulting heart failure. For instance, as demonstrated by the current study, the upmodulation of PTEN signaling was beneficial in the attenuation of CAL-induced ventricular hypertrophy and HF, but the loss of PTEN prevented ventricular hypertrophy in response to the pressure overload by aortic banding in the experiment conducted by Oudit et al. (46). This therefore implies that while the activation of PTEN attenuates myocardial remodeling and HF in the setting of myocardial infarction, the inhibition of PTEN may ameliorate myocardial remodeling in the setting of pressure overload such as hypertension.

Reactive oxygen species, known to cause oxidative stress, are among the mechanisms underlying myocardial damage (41) and were previously demonstrated to cause the oxidation of PTEN, leading to the activation of the PI3K/Akt pathway (47). Therefore, the loss of PTEN activity in this rat HF model could result from reactive oxygen-mediated degradation of PTEN. Since hexarelin treatment decreased oxidative stress in the current study, the antioxidant capacity of hexarelin could be responsible for the increase in PTEN expression indirectly.

We further sought to investigate how hexarelin modulates the known downstream PI3K/Akt-pathway components since its role in cardiac hypertrophic remodeling is well established. Cardiac hypertrophic remodeling response after the activation of PI3K is related to PI3K downstream Akt (48), a known serine/threonine kinase associated with the regulation of different cellular functions for cell survival like protein synthesis, metabolism, proliferation, and glucose uptake. The targeted overexpression of phosphorylated Akt in the heart leads to cardiac hypertrophic remodeling (49) by inhibiting GSK3β to induce cell protein synthetic machinery (50). mTOR is a known serine–threonine protein kinase that becomes activated through the phosphorylation of TSC2 by Akt. The phosphorylation of mTOR is involved in protein synthesis (51,52) and results in cardiac hypertrophic remodeling (53) while the pharmacological inhibition of mTOR is known to reverse cardiac hypertrophic remodeling (54,55). The findings of this study showed that treatment with hexarelin inhibited the phosphorylation of Akt and mTOR accompanying the attenuation of CAL-induced myocardial hypertrophic remodeling and HF. Crackower et al. used mouse cardiac muscle cells to demonstrate that PTEN deletion results in myocardial hypertrophic remodeling and is closely associated with the upsurge in Akt activity (24), suggesting that PTEN activation inhibits Akt and thereby prevents myocardial hypertrophic remodeling. Our results are therefore consistent with their findings.

Several studies have emphasized that apoptosis is associated with myocardial infarction (56–58) and the progression to HF. The expressions of proapoptotic factors like Bax and caspase-3 and an antiapoptotic factor like Bcl-2 are associated with apoptosis (59). We found in our study that hexarelin treatment reduced the expression of proapoptotic factors like Bax and caspase-3 and increased the expression of antiapoptotic Bcl-2, which all seemed to support our Hoechst 33258 and H&E staining results with regard to myocardial cell loss or apoptosis. Another interesting observation in our study is that, although to the best of our knowledge there are no reports on the antiapoptotic action of hexarelin with regard to the Akt/mTOR pathway, the inhibition of apoptosis by hexarelin in our study was accompanied by the downregulation of Akt activation, which seems to be contrary to most of the literature (60). Our findings therefore raise the possibility of the existence of Akt-independent antiapoptotic signal induction by hexarelin, consistent with other Akt-independent antiapoptotic actions by other agents reported in some publications (61,62) and meriting further investigations.

In conclusion, our findings revealed that hexarelin attenuates CAL-induced HF in rats by ameliorating myocardial remodeling, LV dysfunction, and oxidative stress via the upmodulation of PTEN signaling and the downregulation of the Akt/mTOR signaling pathway. Our data provide insights into the mechanisms underlining the possible application of hexarelin in patients with myocardial infarction and HF.

## Acknowledgments

This research was funded by the National Natural Science Foundation of China under grant number 81370342. We would like to express our sincere gratitude to Joyce Massaro for her support by reading over the article.
